# Community and individual level determinants of infant mortality in rural Ethiopia using data from 2016 Ethiopian demographic and health survey

**DOI:** 10.1038/s41598-022-21438-3

**Published:** 2022-10-07

**Authors:** Setegn Muche Fenta, Girum Meseret Ayenew, Haile Mekonnen Fenta, Hailegebrael Birhan Biresaw, Kenaw Derebe Fentaw

**Affiliations:** 1 Department of Statistics, Faculty of Natural and Computational Sciences, Debre Tabor University, Debre Tabor, Ethiopia; 2 Research and Technology Transfer Directorate, Amhara Public Health Institute, P.O. Box 477, Bahir Dar, Ethiopia; 3 Department of Statistics, College of Science, Bahir DarUniversity, Bahir Dar, Ethiopia

**Keywords:** Health care, Medical research, Risk factors

## Abstract

The infant mortality rate remains unacceptably high in sub-Saharan African countries. Ethiopia has one of the highest rates of infant death. This study aimed to identify individual-and community-level factors associated with infant death in the rural part of Ethiopia. The data for the study was obtained from the 2016 Ethiopian Demographic and Health Survey. A total of 8667 newborn children were included in the analysis. The multilevel logistic regression model was considered to identify the individual and community-level factors associated with new born mortality. The random effect model found that 87.68% of the variation in infant mortality was accounted for by individual and community level variables. Multiple births (AOR = 4.35; 95%CI: 2.18, 8.69), small birth size (AOR = 1.29; 95%CI: 1.10, 1.52), unvaccinated infants (AOR = 2.03; 95%CI: 1.75, 2.37), unprotected source of water (AOR = 1.40; 95%CI: 1.09, 1.80), and non-latrine facilities (AOR = 1.62; 95%CI: 1.20) were associated with a higher risk of infant mortality. While delivery in a health facility (AOR = 0.25; 95%CI: 0.19, 0.32), maternal age 35–49 years (AOR = 0.65; 95%CI: 0.49, 0.86), mothers receiving four or more TT injections during pregnancy (AOR = 0.043, 95% CI: 0.026, 0.071), and current breast feeders (AOR = 0.33; 95% CI: 0.26, 0.42) were associated with a lower risk of infant mortality. Furthermore, Infant mortality rates were also higher in Afar, Amhara, Oromia, Somalia, and Harari than in Tigray. Infant mortality in rural Ethiopia is higher than the national average. The government and other concerned bodies should mainly focus on multiple births, unimproved breastfeeding culture, and the spacing between the orders of birth to reduce infant mortality. Furthermore, community-based outreach activities and public health interventions focused on improving the latrine facility and source of drinking water as well as the importance of health facility delivery and received TT injections during the pregnancy.

## Introduction

The infant mortality rate is the most significant public health predictor, as it represents children’s and communities’ access to basic health measures such as vaccination, infectious disease care treatment, and proper nutrition^1^. The Sustainable Development Goals (SDG) child mortality objective seeks to bring an end to preventable deaths of newborns and children under-five years of age by 2030, with all countries aiming to reduce newborn mortality to at least as low as 12 deaths per 1000 live births and under-five mortality to at least as low as 25 deaths per 1000 live births. In 2018, 4.1 million children died worldwide in their first year of life^2–5^.More than one million children die in Africa alone before celebrating their first birthday. These values are approximately equivalent to 2,808 deaths per single day or about two deaths every minute. Since the Millennium Development Goals (MDGs) were adopted, the countries of Sub-Saharan Africa have achieved incredible success and increases in infant survival, but the infant mortality rate in Sub-Saharan Africa is still the highest in the global region^2–5^. Ethiopia has one of the highest infant mortality rates in the world^5,6^. In rural Ethiopia, the infant mortality rate is 62 deaths per 1000 live births. This infant mortality is substantially higher than the SDG targets of 12 deaths per 1000 live births^5,7,8^.

The Ethiopian government is working to reduce child deaths and reforms have been made in recent years. In the rural part of Ethiopia, maternal complications during childbirth, immediate exclusive breast-feeding, birth interval, maternal socioeconomic characteristics, and health service seeking actions, etc., are still major challenges^9,10^. In order to prepare and enforce an initiative and take steps to address the burden of newborn deaths in the rural areas of Ethiopia, identification of the enumeration area specific factors on infant mortality is therefore necessary^4,11^.

Previous studies have concluded that infants born in rural areas are more at risk of death than infants born in urban areas^12–17^. In Ethiopia, infant mortality in rural areas is a major challenge^7,9^. Understanding the causes of infant mortality in rural regions is crucial if we are to reduce Ethiopia's high infant mortality rate. Furthermore, the estimated infant mortality rate in this country was greater in rural areas (62 per 1000 live births) than in urban areas (54 per 1000 live births) and compared to the national average (48 per 1000 live births)^7,18^. Although various studies have been undertaken in Ethiopia to investigate infant death rates and risk factors^16,17,19,20^, little study has been conducted in rural Ethiopia. This lack of epidemiologic study limits our understanding of the determinant to prioritize for evidence-based programming in this high-risk region of infant mortality. Previous studies in Ethiopia on the risk factors for infant mortality were also institutionally focused^21,22^, and only looked at individual-level factors^19,21,22^. However, community-level variables such as the source of drinking water^23^, type of toilet facilities^23^, cluster (enumeration area)^23,24^, and region^23,24^ may all have an effect on infant mortality.

Furthermore, the Ethiopian Demographic and Health Survey (EDHS) used a multistage cluster sampling procedure in which individuals were nested in clusters and infant mortality was correlated with these clusters^7,18^. This violates the assumption of independence, which may introduce a significant bias in programmatic implementation by implying that contextual variables are not taken into account in the study. For example, researchers discovered that geographic access to health care has an impact on infant mortality. Contextual variables, such as the region of respondents, enable researchers to investigate how a wide range of environmental factors may influence health and well-being^25^. To address this, we used a multilevel logistic regression model to examine variables associated with infant mortality at individual and community levels^25,26^. This study aimed to identify individual- and community-level factors associated with infant death in the rural parts of Ethiopia.

## Methods

### Study design and setting

This study was conducted in Ethiopia, which is the second-most populated country in Africa, after Nigeria, and is located in the Horn of Africa. Ethiopia has nine regional states (Tigray, Afar, Amhara, Oromiya, Somali, BenishangulGumuz, Southern Nations Nationalities and People (SNNP), Gambela, and Harari) as well as two city administrations (Addis Ababa and Dire Dawa). We used secondary data from the 2016 EDHS.

### Sampling and data measurements

The 2016 EDHS employed stratified and cluster multistage sampling, with the goal of being representative at the regional and national levels in terms of appropriate demographic and health indicators. In the first stage, 645 clusters (202 in urban areas and 443 in rural areas) were selected using a probability proportional to cluster size and independent selection in each sampling stratum. In the second stage, random samples of 18,008 households were drawn from all identified EAs. A total of 15,683 women aged 15–49 were interviewed. Data was collected from 18 January to 27 June 2016. The sample size for EDHS was determined using a multistage sampling procedure that took into account sampling variation^7^.

### Study variables

#### Outcome variables

The response variable of this study was the status of infant mortality. Infant mortality is defined as the risk of a child dying between birth and their first birthday. This takes a binary outcome; so infant death is classified as either death (1 = if the infant died between birth and their first birthday) or alive (0 = if the infant was alive between birth and their first birthday).

#### Independent Variables

The possible predictor variables associated with infant mortality will be categorized as individual-level factors and community-level factors. These variables were chosen based on previous knowledge and existing literature^12–16,19^ (Table 1).

**Table 1:** Description and measurement of individual- and community-level independent variables.

Variables	Category/Measurement/Definition
**Individual level factors**	
Sex of the child	Male, Female
Birth order	First, 2–3, 4 and above
Age of mother at first birth	< 16, > 16
Types of birth	Single, Multiple
Place of delivery	Home, Health facility
Vaccination of child	Yes, No
Size of child at birth	Larger than average, average, smaller than average
Breastfeeding status	No, Yes
Highest educational level	No education, Primary, Secondary and above
Family size	< 4, > 4
Wealth index	Poor, Medium, Rich
Marital status	Separated , Married
Mothers occupation	Housewife, Employed
Husband education level	No education, Primary, Secondary and above
Number of living children in the household	< 4, ≥ 4
Child has diarrhea in the last week	No, Yes
Preceding birth interval	< 24, > 25
Received TT injections during pregnancy	Not Received, 1–3, ≥ 4
Sex of household head	Male, Female
**Community level factors**	
Place of residence	Urban, Rural
Region	Tigray, Afar, Amhara, Oromia, Somali, Beninshangul-Gumuz, SNNPR, Gambela, Harari, Dire Dawa
Toilet facility use	Categorized as "yes" or "no." If the respondents answered "yes," it means that the household uses one of the following non-shared toilet types: flush/pour flush toilets to piped sewer systems, septic tanks, and pit latrines; ventilated improved pit (VIP) latrines; pit latrines with slabs; and composting toilets
Source of drinking water	Source of drinking water were categorized into Improved sources of drinking water and unimproved sources of drinking water. Improved sources of drinking water includes piped water, public taps, standpipes, tube wells, boreholes, protected dug wells and springs, and rainwater

#### Data management and analysis

The variables were extracted from the BR dataset using SPSS software version 21, and then exported to the statistical software R version 3.5.3 for further analysis. Data were weighted after extraction using sampling weight (v005), main sampling unit (v023), and strata (v021) to account for unequal probability of selection and non-response. Descriptive analysis was done and sample characteristics were presented in frequency and percentages to show the distribution of respondents by the selected variables. The data in EDHS were not flat and were collected using multistage stratified cluster sampling techniques. To draw valid inferences and conclusions, advanced statistical models such as hierarchical modelling, which consider independent variables measured at the individual and community levels, should be used to account for the clustering effect/dependency. A two-level multilevel logistic regression analysis was used to examine the effects of individual- and community-level characteristics on infant death and to determine the extent to which characteristics at the individual and community levels explain enumeration area variations in infant death in rural Ethiopia. The reason for using multilevel logistic regression model was to account for the hierarchical (correlated) structure of the data. The assumption is that infant and their households are nested within enumeration area (communities). This suggests that infant in households with similar characteristics can have different health outcomes when residing in different communities with different characteristics. The log of the probability of infant death was modeled using a two-level multilevel model as follows:

$$ Log\left[ {\frac{{\pi_{ij} }}{{1 - \pi_{ij} }}} \right] = \beta_{0} + \beta_{1} X_{ij} + \beta_{2} Z_{ij} + u_{j} + e_{ij} $$

where, $$i$$ and $$j$$ are the level 1 (individual) and level 2 (community) units, respectively; $$X$$ and $$Z$$ refer to individual and community-level variables, respectively; $$\pi_{ij}$$ is the probability of infant death for the ith women in the *j*th community; the $$\beta$$ indicates the fixed coefficients. Whereas, $$\beta_{0}$$ is the intercept-the effect on the probability of infant death in the absence of influence of predictors; and $$u_{j}$$ showed the random effect (effect of the community on infant death for the *j*th community and $$e_{ij}$$ showed random errors at the individual levels. By assuming each community had different intercept ($$\beta_{0}$$) and fixed coefficient ($$\beta$$), the hierarchical (clustered) data nature and the within and between community variations were taken into account. Four models were fitted to identify community and individual level factors associated with infant death. The first model (Model 1 or empty model) contained no explanatory variables, but was fitted to decompose the total variance into its individual- and community-level components. The second model (Model 2) considered only the individual-level variables in order to examine the individual-level effect. The third model (Model 3) considered only the community-level variables in order to examine the effect of community-level factors on infant death, independent of other factors. The fourth model (Model 4) is the full model that incorporated all the individual and community-level variables into the multilevel analysis. Fitting the final model involved two steps. First, stepwise logistic regression analysis was done to identify the key variables associated with infant death. Second, all the variables selected from the stepwise logistic regression models were incorporated into the multilevel modeling. For the result of fixed effect, odds ratio (ORs) with 95% confidence intervals (CIs) was used to declare statistical significance. The *P*-value ≤ 0.05 has been considered as statistically significant. The measures of variation (random-effects) were summarized using ICC, Median Odds Ratio (MOR) and proportional change in variance (PCV) to measure the variation between enumeration areas (clusters). ICC is a measure of within-cluster variation, the variation between individuals within the same cluster, and it was calculated using the formula: $$ ICC = ~\frac{{V_{A} }}{{V_{A}  + {\raise0.7ex\hbox{${\pi ^{2} }$} \!\mathord{\left/ {\vphantom {{\pi ^{2} } 3}}\right.\kern-\nulldelimiterspace} \!\lower0.7ex\hbox{$3$}}}} = ~\frac{{V_{A} }}{{V_{A}  + 3.29}} $$ , where $${V}_{A}$$ is the estimated variance in each model, which has been described elsewhere^27^. The total variation attributed to individual or/and community level factors at each model was measured by the proportional change in variance (PCV), which was calculated as: $$CV= \frac{{V}_{A}-{V}_{B}}{{V}_{A}}$$ , where $${V}_{A}$$ = variance of the initial model, and $${V}_{B}$$ = variance of the model with more terms^25^. The MOR is the median odds ratio between the individual of higher propensity and the individual of lower propensity when comparing two individuals from two different randomly chosen clusters and it measures the unexplained cluster heterogeneity, the variation between clusters by comparing two persons from two randomly chosen different clusters. It was computed using the formula:

$MOR=\mathrm{exp}(\sqrt{2*{V}_{A}*0.6745} )\approx \mathrm{exp}(0.95\sqrt{{V}_{A}} )$, where ${V}_{A}$ is the cluster level variance^25,27^. The MOR measure is always greater than or equal to 1. If the $MOR$ is 1, there is no variation between clusters ^27^. The generalized variance-inflation factor (GVIF) test was used to check for multicollinearity; the findings showed that there no multicollinearity because the GVIF for each variable was less than 5. Model comparison was done using Deviance Information Criteria (DIC), Akaike’s Information Criterion (AIC) and Bayesian’s Information Criterion (BIC). The model with the smallest value of the information criterion was selected as the final model of the analysis^27^.

#### Ethical Issues.

Publicly available EDHS 2016 data was used for this study. Informed consent was taken from each participant, and all identifiers were removed.

#### Confirmation of methods

Author(s) confirm that all methods were carried out in accordance with relevant guidelines and regulations in the manuscript.

## Results

### Socio-demographic and obstetric characteristics of the study respondents

The total number of infants participated in this study was 8667. 6568 (73.8%) of them were born at home, and 4467 (51.1%) of them were males. 6509 (75.1%) of mothers were housewives and 6267 (72.3%) had no formal education. About 8439 (97.4%) infants were born as singletons and 5625 (64.9%) infants were born into low-income household. The majority; 7129 (82.3%) of mothers have used improved sources of drinking water. About 5350 (61.7%) of mothers did not have visits during pregnancy and 5857 (67.6%) of women did not receive tetanus injection during pregnancy. 4675 (53.9%) of mothers had 3 or above ever-born children and only 458 (5.3%) infants were not breastfeeding at all (Table 2).

**Table 2:** Socio-demographic and obstetric characteristics of the study respondents EDHS 2016.

Individual- and community-level characteristics	Frequency	Percentage
**Age of mother at first birth**		
< 16	2340	27.0
> 16	6327	73.0
**Number of living children in the household**		
< 4	3992	46.1
> 4	4675	53.9
**Sex of child**		
Male	4467	51.5
Female	4200	48.9
**Place of delivery**		
Home	6568	75.8
Health facility	2099	24.2
**Birth order number**		
First order	1492	17.2
2–4	3682	42.5
> 5	3493	40.3
**Type of birth**		
Single birth	8439	97.4
Multiple birth	228	2.6
**Child has diarrhea in the last week**		
No	7137	82.5
Yes	1530	17.7
**Size of child at birth**		
Larger than average	2525	29.1
Average	3536	40.8
Smaller than average	2606	30.1
**Breastfeeding status**		
No	458	5.3
Yes	8209	94.7
**Preceding birth interval in month**		
< 24	2384	27.5
> 25	6283	72.5
**Received TT injections during pregnancy**		
Not Received	5857	67.6
1–3	2453	28.3
> 3	357	4.1
**Vaccination of child**		
Yes	3408	39.3
No	5259	60.7
**Number of ANC visits**		
No visit	5350	61.7
1–3	1714	19.8
> 4	1603	18.5
**Mothers age**		
15–24	2133	24.6
25–34	4927	56.8
35–49	1607	18.5
**Mothers educational status**		
No education	6267	72.3
Primary	2041	23.5
Secondary and above	359	4.1
**Family size**		
< 4	2189	25.3
> 4	6478	74.7
**Wealth index**		
Poor	5625	64.9
Middle	1417	16.3
Rich	1625	18.7
**Marital status**		
Separated	498	5.7
Married	8169	94.3
**Mothers occupation**		
Housewive	6509	75.1
Employed	2158	24.9
**Husbands educational status**		
No education	4578	52.8
Primary	2748	31.7
Secondary and above	1341	15.5
**Sex of household head**		
Male	7038	81.2
Female	1629	18.8
**Region**		
Tigray	864	10.0
Afar	968	11.2
Amhara	888	10.2
Oromia	1506	17.4
Somali	1204	13.9
Benishangul	829	9.6
SNNPR	1182	13.6
Gambela	539	6.2
Harari	404	4.7
Dire Dawa	283	3.3
**Source of drinking water**		
Improved	1538	17.7
Unimproved	7129	82.3
**Improved toilet facility**		
Yes	4026	46.5
No	4641	53.5

*ANC* Antenatal Care, *SNNPR* Southern nations, nationalities, and people region, *TT* Tetanus Toxoid.

### Factors associated with the infant death

The result of the multilevel logistic regression model was summarized in Table 2. The model selection result indicated that model IV was a better fit to the data as compared to other reduced models, since it has the smallest AIC, BIC, and deviance statistic. The final selected model (model IV) showed that antenatal care visit, preceding birth interval, number of TT injections, education level of the mother, family size, vaccination of the child, contraceptive use, child twin, place of delivery, diarrhea status, size of child at birth, marital status, number of living children, breastfeeding, region, water source, and latrine facility type were found to have a statistically significant association with infant mortality (Table 3 and Table 4).

**Table 3:** multilevel logistic regression analysis for risk factors of infant mortality in rural Ethiopia, 2016.

Individual- and community-level characteristics	Model I	Model II	Model III	Model IV
	AOR (95% CI)	AOR (95% CI)	AOR (95% CI)	AOR (95% CI)
**Number of ANC visits**				
No visit		1		
1–3		0.605 (0.509, 0.720)*		0.749 (0.623, 0.901)*
> 4		0.610 (0.511, 0 .729)*		0.787 (0.645, 0.961)*
**Preceding birth interval in month**				
< 24		1		1
> 24		0.825 (0.639, 1.065)*		0.724 (0.624, 0.839)*
**Number of received TT injections during pregnancy**				
Not received		1		1
1–3		0.041 (0.032, 0.053)*		0.040 (0.031, 0.051)*
> 4		0.045 (0.027, 0.075)*		0.043 (0.026, 0.071)*
**Types of birth**				
Single birth		1		1
Multiple birth		4.435 (2.178, 9.032)*		4.350 (2.179, 8.685)*
**Place of delivery**				
Home		1		1
Health facility		0.206 (0.160, 0.267)*		0.249 (0.193, 0.321)*
**Child vaccination**				
Yes		1		1
No		2.205 (1.915, 2.539)*		2.033 (1.745, 2.370)*
**Child has diarrhea in the last week**				
Yes		1		
No		0.758 (0.569, 1.010)*		0.719 (0.597, 0.866)*
**Size of child at birth**				
Larger than average		1		1
Average		0.894 (0.766, 1.042)		0. 910 (0.777, 1.067)
Smaller than average		1.267 (1.084, 1.481)*		1.290 (1.096, 1.519)*
**Breastfeeding**				
No		1		1
Yes		0.304 (0.240, 0.384)*		0.329 (0.260, 0.418)*
**Mothers educational status**				
No education		1		1
Primary		0.761 (0.585, 0.990)*		0.859 (0.739, 0.998)*
Secondary and above		0.778 (0.441, 1.370)		0.927 (0.676, 1.270)
**Family size**				
< 4		1		1
> 4		1.585 (1.173, 2.142)*		1.623 (1.193, 2.206)*
**Marital status**				
Separated		1		1
Married		1.069 (0. 816, 1.401)		0.670 (0.485, 0.925)*
**Number of living children**				
< 3		1		1
> 3		1.386 (1.021, 1.882)*		1.529 (1.052, 2.223)*
**Region**				
Tigray			1	1
Afar			1.243 (0.859, 1.800)	2.564 (1.466, 4.487)*
Amhara			1.747 (1.223, 2.496)*	3.326 (2.064, 5.361)*
Oromia			2.743 (1.977, 3.806)*	12.070 (7.584, 19.21)*
Somali			2.429 (1.738, 3.395)*	7.653 (4.598, 12.739)*
Benishangul-gumz			1.992 (1.374, 2.887)*	4.171 (2.501, 6.955)*
SNNPR			1.715 (1.209, 2.434)*	4.083 (2.561, 6.509)*
Gambela			1.275 (0.840, 1.936)	5.436 (2.900, 10.188)*
Harari			2.169 (1.392, 3.379)*	7.067 (3.679, 13.575)*
Dire Dawa			1.755 (1.043, 2.951)*	3.330 (1.647, 6.732)*
**Source of drinking water**				
Protected			1	1
Unprotected			1.262 (1.045, 1.525)*	1.400 (1.087, 1.802)*
**Improved toilet facility**				
Yes			1	1
No			1.242 (1.074, 1.437)*	1.621 (1.201, 2.187)*

1 reference category for categorical variable and *reference *P*-value < 0.0001.

*ANC* Antenatal Care, *AOR* Adjusted odds ratio, *SNNPR* Southern nations, nationalities, and people region, *TT* Tetanus Toxoid.

**Table 4:** Measure of variation on individual and community level risk factors of infant death in rural Ethiopia, EDHS 2016 dataset.

Measure of variation	Model I (Null model)	Model II	Model III	Model IV (Full model)
Variance (SE)	1.615(0.140)*	0.313 (0.054)*	0.876(0.141)*	0.199 (0.044)*
PCV (%)	Reference	80.62	45.76	87.68
ICC (%)	32.93	8.69	21.03	5.70
MOR	3.344	1.701	2.433	1.528
**Model fit statistics**				
DIC (-2log likelihood)	7196.184	2591.376	7127.01	**2461.344**
AIC	7200.183	2649.376	7153.01	**2541.345**
BIC	7214.318	2827.259	7244.884	**2786.7**

*reference *P*-value < 0.0001.

*AIC* Akaike’s information criterion, *BIC* Bayesian’s information criterion, *DIC* Deviance information criterion, *ICC* Intra-cluster correlation, *MOR* Median odds ratio, *SE* Standard Error, *PCV* Proportional change in variance.

### Individual-level factor

The odds of infant death among mothers who had four or more ANC visits during their pregnancy was 0.787 (AOR = 0.787, 95%CI: 0.645, 0.961) times lower as compared to mothers who had no ANC visits during their pregnancy. The odds of infant death for infants with preceding birth interval less than 2 years were increased by 27% (AOR = 0.724, 95%CI: 0.624, 0.839) compared to infants with preceding birth interval more than 2 years. The odds of infant death among multiple birth were 4.35 (AOR = 4.350; 95% CI, 2.179, 8.685) times higher as compared to singletons. Infants born to mother who attained primary education had 0.859 (AOR = 0.859; 95% CI: 0.739, 0.998) times lower likelihood of infant death than infants whose mother did not have formal education. The odds of infant death who are born at the health facility were 0.249 (AOR = 0.249; 95% CI, 0.193, 0.321) times lower as compared to children who are born at home. Infants who had not received vaccination had a 2.033 (AOR = 2.033; 95% CI, 1.745, 2.370) times higher risk of death than infants who had received vaccination. The odds of infant death were 1.29 (AOR = 1.290; 95% CI, 1.096, 1.519) times higher in infants born with a small birth size compared to infants born with a large birth size. As compared to separated mothers, married mothers had 0.670 (AOR = 0.670; 95% CI, 0.485, 0.925) times lower odds of infant death. The odds of infant death among mothers who received TT injections 4 and above times during pregnancy was 0.043 (AOR = 0.043, 95%CI: 0.026, 0.071) times lower as compared to mothers who did not receive TT injection during pregnancy. Families with five or more members were 1.623 times (AOR = 1.623; 95% CI: 1.193, 2.206) more likely to lose an infant than families with four or fewer members. The risk of infant death was reduced by 67.1% (AOR = 0.329; 95%CI: 0.260, 0.418) in mothers who breastfed compared to mothers who did not breastfeed (Table 2).

#### Community level factor

Infants living in Afar (AOR = 2.564: 95%CI: 1.466, 4.487), Amhara (AOR = 3.326; 95%CI: 2.064, 5.361), Oromia(AOR = 12.070; 95% CI: 7.584, 19.21), Somali(AOR = 4.171; 95% CI: 2.501, 6.955), SNNPR (AOR = 4.083; 95%CI: 2.561, 6.509) and Harari (AOR = 7.067; 95%CI: 3.679, 13.575) regional state were more likely die as compared to infants living in Tigray regional state. Infants from households without access to a latrine had 62.1% (AOR = 1.621; 95%CI: 1.201, 2.187) higher odds of death compared with infants from households that had an improved latrine facility. The probability of infant death for women those use unprotected source of water was increased by 40% (AOR = 1.400; 95%CI: 1.087, 1.802) as to women use protected water sources (Table 3).

### Random effect (a measure of variation)

In Table 4, the results of the random-effects model are given. There were varying infant mortality rates across clusters (communities). Significant variations in infant mortality at the community level were seen in the results of the null model (Model I). The findings show that 32.93% of intra-class correlations (ICCs) were correlated with infant mortality at the community level. After adding both the individual-level and community-level factors in the model (Model IV), there is a significant variation of infant mortality across communities or clusters. About 87.68% of infant mortality in clusters was explained in the full model. Moreover, the MOR confirmed that infant mortality was attributed to community-level factors. In the null model, the MOR for infant mortality was 3.344; this showed that the difference between communities (clustering) was 3.344 times greater than the reference (MOR = 1). When all variables were included in the model, the unexplained community variation in infant deaths was reduced to an MOR of 2.04. This indicated that when all factors are considered, the effects of clustering are still statistically significant in the full models (Table 4).

## Discussion

This study aimed to investigate modifiable risk factors in rural Ethiopia for infant mortality. In addition, it explored the variation of rural infant mortality in the enumeration area that has not been studied so far. From the 2016 EDHS data, 8667 rural infants nested in 443 clusters were included in the analysis. The infant mortality rate in rural Ethiopia in 2016 was 62 deaths per 1000 live births^7^. This death rate is higher than 54 deaths per 1000 live births in urban Ethiopia^7^. This may be due to the prevalence in rural settings of weak infrastructure, low-economic classes, and restricted flow of information, where the level of risk is estimated to be high. The random effect model showed that both individual and community-level factors accounted for about 87.68% of the variation observed for infant mortality. This finding was in line with the study conducted in Ethiopia^24^.

A lower risk of infant death has been associated to having a wealthy household. Previous studies in Ethiopia^20^, Bangladesh^15^, rural district in Indonesia^28^ and Nigeria^29^ showed that infant mortality was negatively associated with household income. Infants from high-income families would be able to meet basic needs and services including health care, quality of life, water quality, and sanitation^25^. Compared to short birth intervals, long birth intervals were associated with a lower risk of infant death, and the risk of infant death decreased as the previous birth interval increased. This was supported by the study findings in Bangladesh^15^, Ethiopia^19,20,30^ and Tanzania^31^. The reason behind this could be shorter preceding birth intervals are linked to an increased risk of preterm birth, low birth weight, and IUGR for subsequent births. Furthermore, women had less time to recover from previous births and were less able to provide sustenance for their children, potentially increasing the risk of infant mortality.

The study result showed that; the odds of infant death among mothers who received TT injections during pregnancy was lower as compared to mothers who did not receive TT injection during pregnancy This finding was in agreement with a study done in Ethiopia^11,17,25^ and rural district in Indonesia^28^ . This could be due to the fact that TT injection produces protective antibodies against infant tetanus^11^.

The women’s education level was an important socio-economic predictor of infant death. Educated mothers had lower infant mortality than uneducated mothers. This is similar to studies done in Bangladesh^15^, Ethiopia^19,32^, Nigeria^29,33^ and Brazilian^34^, which found the infant death rate decreased with an increase in the level of education of the mother. Educated mothers are more likely to be conscious of nutrition, use of contraceptives to space births, and awareness of childhood diseases and care.

This study found that ANC visit was a significant predictor of infant mortality. Women who did not have an ANC visit during pregnancy had a greater risk of infant death than those who did. This finding is consistent with the study conducted in Ethiopia^11^, rural district in Indonesia^28^, Nepal^35^ and Pakistan^36^. This could be because antenatal care visits provide health benefits such as iron, folic acid, and tetanus vaccines, which may reduce the risk of infant mortality. Furthermore, ANC provides mothers and newborns with the opportunity to undergo various interventions like as anti-D, childhood vaccines, and nutritional supplementation^37^.

Infant mortality was positively associated with multiple births among infants. The odds of infant death among multiple births were higher as compared to singletons. This finding is in agreement with a study from Ethiopia^19,20,24^ showed that the risk of infant deaths due to multiple births is very high. Infants born at the health center were at lower risk than infants born at home. Multiple births are regarded to put a strain on a family's finances, affecting the infant's nutrition and health care. Multiple births have been related to a higher rate of negative prenatal outcomes, such as premature birth and low birth weight.

Place of delivery was a significant predictor of infant mortality. Infants born health facilities had a lower risk of death when compared to those born at home. This finding was in agreement with a study done in Ethiopia^4^ rural district in Indonesia^28^and Nigeria^29^. This outcome could be explained by the fact that the place of delivery is required to promote the health of women and fetuses by lowering birth complications.

Child vaccination was significantly correlated with infant mortality. Non-vaccinated infant had a greater risk of death than those who had been vaccinated. It is supported by other findings in Ethiopia^17,38^. The possible reason could be that vaccines can prevent infectious diseases that once killed or harmed many infants. Breastfeeding was found to be significantly associated with infant death. Currently breastfeeding infants have fewer chances of dying with infants than non-breastfed infants. This is also consistent with previous research conducted in Ethiopia^17,20^ and Nepal^39^. This may be because breastfeeding may protect babies from infectious diseases since the fluids from the breast are high in antibodies and white cells.

The results of this study also indicated that infant deaths are significantly impacted by family size. The likelihood of infant death increased significantly as family size increased. Similar findings were also found in Ethiopia^20^. The potential explanation for this may be that there are too many siblings residing at home, which may result in baby care that is insufficient and inappropriate. The study also revealed that number of living children in the household is an important variable affecting infant mortality. As the number of living children in the household increased the risk of infant mortality increased significantly. A study from Ethiopia^17^, Bangladesh^15^ and rural district in Indonesia^28^ consistently reported that infant mortality increase with increase in the number of living children.

Separated women had a higher risk of infant mortality than married women. This finding is in agreement with a study from Rwanda^40^ and United States^41^ .This could be due to socioeconomic issues, cultural norms, and the lifestyle consequences of single women. Similarly, infants born with a small birth size had a higher risk of infant mortality than infants born with a large birth size. This result is in line with the previous findings in Ethiopia^19,20^, Bangladesh^14,42^ and Indonesia^43^. Poor nutritional status may have an impact on size at birth, which may have an impact on the risk of newborn mortality^19^.

The studies also revealed that infant mortality was influenced by geographic location. Infants born in the regional states of Afar, Amhara, Oromia, Somalia, SNNPR, Benishangul-gumz, Gambela, Dire Dawa, and Harari were more likely to die than those born in Tigray. It is supported by other findings in Ethiopia^24^, Bangladesh^13^ and Nigeria^29,33^. The potential explanation for this may be the regional disparities in socioeconomic status, health-care coverage, and other amenities.

Women who drank from an unimproved source had a greater risk of infant death than women who drank from a safe and protected source. This finding is in line with studies done in Bangladesh^15^, Pakistan^44^ and Nigeria^45^. This could be because protected sources of drinking water are less likely to be polluted and less likely to prevent the spread of water-borne diseases like infections and cholera. Infants born into families without access to a latrine died at a higher rate than those born into families with superior latrine facilities. It is supported by other findings in Pakistan^44^ and Nigeria^45^ . This may be because access to modern sanitation facilities such as flush toilets is reducing diarrhea prevalence and ultimately decreasing the infant deaths^23^.

### Strengths and limitation

EDHS are a national representative household survey with a high response rate and the findings are generalized to the national populations. The study focused on national survey data that provides policy makers and program managers insight into the implementation of effective intervention strategies both at national as well regional level. In addition, this study applied multilevel modeling to accommodate the EDHS data hierarchical nature. Because of the cross-sectional nature of the data, it is difficult to measure the causal effect and it is not possible to know if the data depends on time or not.

## Conclusion

Infant mortality in rural Ethiopia is higher than the national average. This study has demonstrated the importance of both individual and community level factors in explaining enumeration area variations in infant death. This study indicated that antenatal visit, preceding birth interval, number of TT injections, education level of the mother, family size, vaccination of child, contraceptive use, child twin, place of delivery, diarrhea status, size of child at birth, marital status, number of living children, breastfeeding, region, water source, and latrine facility type were found to have a statistically significant association with infant death. The findings suggest that the government and other stakeholders should mainly focus on multiple births, unimproved breastfeeding culture, and the spacing between the orders of birth to reduce infant mortality. Community-based outreach activities and public health interventions focused on improving the latrine facility and source of drinking water as well as the importance of health facility delivery and received TT injections during the pregnancy. The findings of this study may provide a national perspective on the factors that contribute to infant mortality in rural Ethiopia. Finally, we advised policymakers and governments to prioritize community-level factors over individual factors in order to meet the SDG goals and targets by the end of 2030.

## Data Availability

This study used EDHS 2016 child data set and extracted the outcome and explanatory variables. Data is publicly available online from (https://dhsprogram.com/Data/). Correspondence and requests for data and materials should be addressed to S.M.

## Abbreviations

AIC: Akaike’s information criterion
AOR: Adjusted odds ratio
CI: Confidence intervals
CSA: Central Statistical Agency
DIC: Deviance information criterion
EAs: Enumeration areas
EDHS: Ethiopian demographic and health survey
ICC: Intra-cluster correlation
MOR: Median odds ratio
PCV: Proportional change in variance
SNNPR: Southern Nations, Nationalities, and People Region
TT: Tetanus Toxoid
WHO: World Health Organization
